# The response of *Plasmodium falciparum* to isoleucine withdrawal is dependent on the stage of progression through the intraerythrocytic cell cycle

**DOI:** 10.1186/s12936-020-03220-w

**Published:** 2020-04-08

**Authors:** Kyle Jarrod McLean, Marcelo Jacobs-Lorena

**Affiliations:** 1 Department of Molecular Microbiology and Immunology, Johns Hopkins Bloomberg School of Public Health, Johns Hopkins Malaria Research Institute, Baltimore, MD USA; 2 Department of Biological Engineering, Massachusetts Institute of Technology, Cambridge, MA USA

**Keywords:** *Plasmodium falciparum*, Cell cycle, Checkpoint, Isoleucine deprivation

## Abstract

**Background:**

A previous study reported that the malaria parasite *Plasmodium falciparum* enters an altered growth state upon extracellular withdrawal of the essential amino acid isoleucine. Parasites slowed transit through the cell cycle when deprived of isoleucine prior to the onset of S-phase.

**Methods:**

This project was undertaken to study at higher resolution, how isoleucine withdrawal affects parasite growth. Parasites were followed at regular intervals across an extended isoleucine deprivation time course across the cell cycle using flow cytometry.

**Results:**

These experiments revealed that isoleucine-deprived parasites never exit the cell cycle, but instead continuously grow at a markedly reduced pace. Moreover, slow growth occurs only if isoleucine is removed prior to the onset of schizogony. After S-phase commenced, the parasite is insensitive to isoleucine depletion and transits through the cell cycle at the normal pace.

**Conclusions:**

The markedly different response of the parasite to isoleucine withdrawal before or after the onset of DNA replication is reminiscent of the nutrient-dependent G1 cell cycle checkpoints described in other organisms.

## Background

**Fig. 1:**
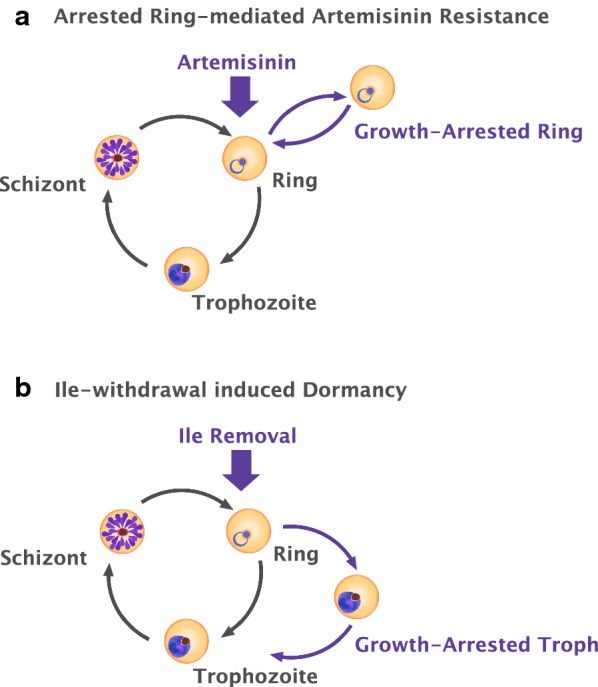
Models for *Plasmodium falciparum* survival after artemisinin treatment and isoleucine deprivation. **a** Upon artemisinin treatment, ring stage parasites exit the cell cycle and enter a programmed state of growth arrest to avoid drug-induced cellular damage, and resume growth once drug levels have subsided. While initial observations appeared to support this model, recent studies of artemisinin-resistant field isolates suggest an alternate model. **b** Upon withdrawal of extracellular isoleucine in vitro, *Plasmodium falciparum* initially slows growth, and may exit the cell cycle and arrest at the trophozoite stage prior to DNA replication. Isoleucine-deprived parasites can remain viable for several days and resume growth again upon isoleucine resupplementation

The frontline anti-malarial drug artemisinin is exhibiting decreased efficacy in clearing *Plasmodium falciparum* from the bloodstream in regions of South East Asia. While there has yet to be widespread clinical failure, parasite isolates that display delayed clearance, detectable in the blood as ring stages hours after artemisinin therapy, have been referred to as artemisinin resistant [1]. The observation that this resistance phenotype manifests as the persistence of a defined stage of the cell cycle (ring), and that artemisinin is most effective when parasites are actively metabolizing haemoglobin in later stages of the cell cycle [2], has led several authors to propose a dormancy model of artemisinin resistance [3–7]. As per this model, haemoglobin non-digesting ring stage parasites exit the cell cycle upon artemisinin treatment and assume a programmed arrested state of low metabolic activity for a prolonged period, until drug concentration has decayed (Fig. 1a). The implications of such ability are important. A programmed state of low metabolic activity could render the parasite insensitive to a broad range of inhibitors, as has been the case for the dormant stages of the bacterial pathogen *Mycobacterium tuberculosis* [8] or the exoerythrocytic stages of other human malaria parasites [9], limiting an already restricted pool of available therapeutics.

Many eukaryotic organisms use phenotypic dormancy to overcome unfavourable environmental conditions. For human cells in culture, removal of serum growth factors causes G1 cells to exit the cell cycle and enter a state of quiescence [10]. Similarly, in haploid stages of the yeast *Saccharomyces cerevisiae*, starvation of nitrogen or carbohydrates during the G1 phase causes an arrest of the mitotic cycle and entry into a dormant state that can be maintained for a prolonged period with little loss of viability [11]. In a recent study, Babbitt et al. reported that *P. falciparum* enters a potentially analogous state when deprived of an extracellular source of the essential amino acid isoleucine [12].

During the intraerythrocytic life cycle, *P. falciparum* acquires most of its amino acid supply through the import and digestion of its host’s haemoglobin. However, because human haemoglobin does not contain isoleucine and the parasite cannot synthesize it de novo, it must obtain isoleucine from the extracellular environment (human serum in vivo or culture medium in vitro). Human blood isoleucine concentrations under certain conditions can drop well below the levels needed to support *P. falciparum* growth in culture [13, 14], which would be lethal for the parasite without an adaptive mechanism. When deprived of extracellular isoleucine, Babbitt et al. reported that the parasite dramatically slows its cell cycle and fails to commence DNA synthesis after 72 h of withdrawal. The parasite can remain in this state of low metabolic activity for upwards of several days, and then resume growth with little loss of viability upon isoleucine resupplementation. In contrast, glucose starvation leads to rapid parasite death within a few hours [12]. An interpretation of this observation is depicted in Fig. 1b.

While the isoleucine deprivation response described by Babbitt et al. does not completely recapitulate the dormancy model for resistance to artemisinin, it is the first clear demonstration that the parasite can modulate its growth in response to environmental changes. A better understanding of this process may reveal underlying molecular processes shared between the starvation and drug resistance responses.

This work revisits, at a high resolution, the response of *P. falciparum* to isoleucine deprivation in vitro. While no evidence for a true cell cycle arrest or dormancy was found, the parasite does enter a state of slow growth upon isoleucine withdrawal. Furthermore, like the nutrient-dependent G1 checkpoint responses in other eukaryotes, entrance into this slow growth state is cell cycle stage-dependent, suggesting an analogous checkpoint may exist in the *P. falciparum* cell cycle.

## Methods

### Parasite strains

The NF54 clone used for most experiments was produced by and obtained from the lab of David A. Fidock at Columbia University [15]. The Hsp101-DD conditional PTEX export 3D7 clone [16] was produced by and obtained from the lab of Dan Goldberg at Washington University.

### Parasite culture and media

Parasites were cultured using standard methods [17] in flasks gassed with a mixture of 90% N_2_, 5% CO_2_, 5% O_2_ (Airgas, #Z03NI9022000033). Normal culture medium consisted of RPMI 1640 with l-glutamine and 25 mM HEPES (Corning, #10-041-CV), with 5.0 g/L Albumax II Lipid-Rich BSA (ThermoFisher Scientific, #11021029), 3.7 mM hypoxanthine, and 50 μg/mL gentamicin. The isoleucine deficient medium consisted of 10.3 g/L RPMI 1640 Ile-Dropout medium (US Biologicals, #R9014), supplemented with 2.0 g/L NaHCO3, 6.0 g/L HEPES, 5.0 g/L Albumax II, 3.7 mM hypoxanthine, and 50 μg/mL gentamicin. Trimethoprim was obtained from Sigma Aldrich (#T7883) and used at a final concentration of 10 μM.

### Flow cytometry

For each sample to be analysed, 1 mL of parasite culture was pelleted and resuspended in 1 mL of PBS containing 4% w/v paraformaldehyde. Samples were rocked at 4 °C for 20–30 h of fixation. Samples were then pelleted and resuspended in PBS containing 0.1% v/v Triton-X 100 and rocked at room temperature for 1 h for permeabilization. This process was then repeated three additional times with PBS (without Triton-X 100) to remove as much haemoglobin as possible. Samples were then diluted approximately 100-fold into normal culture medium containing 1× SYBR Green I (ThermoFisher Scientific, #S7563) and analysed on a FACSCalibur (BD Biosciences) flow cytometer. The resulting data was further analysed using FLOWJO 10.0.7 analysis software. To determine the DNA content of infected red blood cells, an asynchronous culture was stained for DNA and analysed by flow cytometry to determine the median fluorescence intensity of the 1C parasite population. The higher DNA content values were called based on cell populations having an intensity peak at multiples of the median 1C fluorescence intensity obtained during the same flow cytometry session (e.g. a cell population with a median fluorescent intensity at twice the 1C intensity would be called as ‘2C’).

### Statistical analysis

All statistical analyses were performed using R version 3.2.3 (2015-12-10) [18]. T-tests, linear and logistic regressions, and some figures were generated using R base functions. The *drc* package [19] was used for determining the 50% effect points of logistic models, and the *ggplot2* package [20] was used for the production of several figures.

## Results

### *Plasmodium falciparum* completes the cell cycle when deprived of extracellular isoleucine

Babbitt et al. reported that when young rings are deprived of extracellular isoleucine the parasites enter a physiological state reminiscent of hibernation or aestivation in higher animals: a reversible state of low-metabolic activity and high resource conservation [12]. When resupplemented with isoleucine, parasites immediately resumed normal growth, displaying only modest losses in viability after 72 h of withdrawal. Over the course of 72 h of isoleucine deprivation, the parasite’s progression through the cell cycle was drastically retarded, advancing at 40% of the pace of control cultures and never progressing past haemozoin-containing, pre-S-phase trophozoites. The Babbitt et al. experiments were repeated to verify that the phenotype holds for the NF54 subclone of *P. falciparum* used in the authors’ laboratory.

**Fig. 2:**
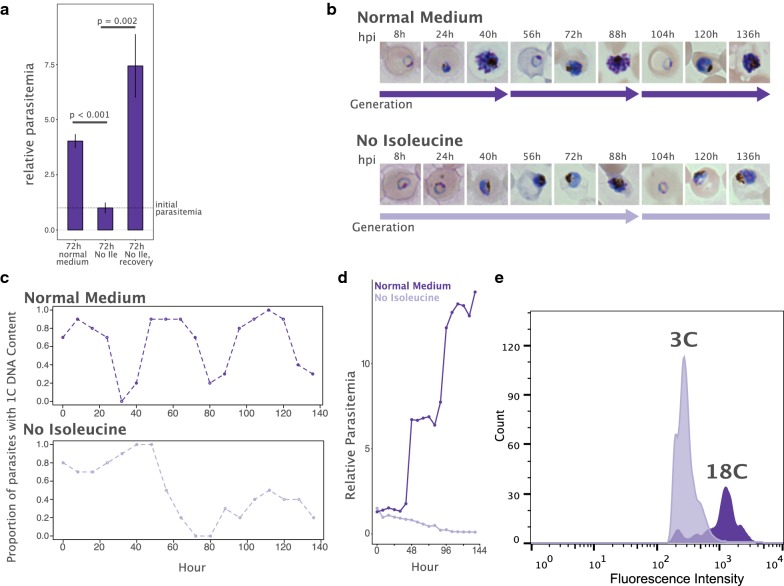
In the absence of extracellular isoleucine, *Plasmodium falciparum* completes the cell cycle at a retarded rate. **a** Synchronous young ring NF54 parasites were cultured in normal culture medium, or culture medium lacking isoleucine (No Ile) for 72 h. “Recovery” denotes parasites that were transferred back to normal medium after deprivation for 72 h of regrowth (t-test, error bars represent standard error, n = 3). **b** Synchronous cultures of NF54 parasites were initiated with Percoll-isolated late schizonts in either normal medium or medium lacking isoleucine. Progression of each culture was tracked every 8 h for 136 h. Images are representative of the major forms observed at each time point. “Generation” represents the time required to transition from ring through the cell cycle to ring again. **c** Flow cytometry was used to track the DNA content of the cultures over the 136-h time course. Dotted-line plots represent the proportion of parasites with a 1C DNA content. Isoleucine-deprived parasites proceed through the cell cycle at approximately half the rate as those in normal medium. **d** Parasitaemia of cultures across the 136-h incubation. **e** Flow cytometry was used to determine the average DNA content of SYBR Green I-stained parasites at the first schizont-enriched time point (40 h for normal medium; 88 h for isoleucine-deprived medium; cf. Fig. 2b) for each treatment across the 136-h time course. “C” refers to the number of genome complements per cell

As observed by Babbitt et al. [12], young rings (~ 6 h post-infection or hpi) washed thoroughly with PBS and transferred to culture medium lacking isoleucine remained at the starting parasitaemia after 72 h, whereas control parasites (standard RPMI 1640 with 382 μM Ile) completed a cell cycle and re-invaded red blood cells over the same time period (Fig. 2a). When the isoleucine-deprived parasites were resupplemented with complete medium for 72 h, they displayed growth similar to the control cultures that had never been deprived (Fig. 2a). Thus, the isoleucine response described by Babbitt et al. is robust and reproducible in the NF54 clone used in the authors’ laboratory.

Over the course of the experiments, parasites with greater than the 1C haploid DNA content were detected in cultures deprived of isoleucine for over 48 h, suggesting that, despite growth retardation, some parasites were able to commence DNA synthesis in the absence of extracellular isoleucine. To investigate this in more detail, isoleucine-dropout and isoleucine-normal cultures from late-stage, Percoll-isolated schizonts were initiated and tracked for their development over the ensuing 6 days.

Control parasites developed as expected, with schizonts appearing in Giemsa-stained smears roughly every 45 h, followed by an increase in parasitaemia with each generation (Fig. 2b, d). DNA content analysis by flow cytometry showed that the proportion of parasites containing 1C DNA peaked approximately every 45 h (Fig. 2c). Isoleucine-deprived parasites also progressed through the cell cycle, albeit at a much-reduced rate (Fig. 2b). Schizonts first became detectable at around 90 h of incubation, and the first rings of the second generation were observed at 100 h of incubation. DNA content tracking showed that 1C parasites peak at roughly 90 h, corresponding to about 50% reduction in the pace of the cell cycle (Fig. 2c). Thus, isoleucine-deprived parasites do not arrest prior to S-phase to assume a dormant state, but instead continuously progress through the cell cycle at a retarded pace. This continued growth during isoleucine withdrawal may account for why parasites that were deprived and then resupplemented, sometimes display higher growth than normal medium controls upon recovery (Fig. 2a). After 72 h of slow growth in media lacking isoleucine, the parasites would have reached S-phase. Upon return to normal media, the 72-h recovery period would allow these late stage parasites to egress and reinvade twice.

While control parasites increased in parasitaemia with each generation, the isoleucine-deprived parasites decreased in numbers at a modest, but steady rate over the course of a 6-day incubation (Fig. 2d). This suggests that, despite completing the cell cycle and re-invading at roughly 4-day intervals, the overall viability of the slow growing parasites was affected by exogenous isoleucine withdrawal. This agrees with the observation of Babbitt et al. of diminished recovery upon isoleucine resupplementation as a function of increased withdrawal time. The mean DNA content of parasites at schizont-enriched time points was substantially lower for isoleucine-deprived parasites compared to controls (Fig. 2e). This suggests that either isoleucine-deprivation produced schizonts with a lower average merozoite count, decreasing the growth potential of each cell, or that a majority of cells experienced catastrophic failure early in schizogony and produced no viable progeny. The low number of schizonts at these timepoints prevented experiments distinguishing these possibilities.

### The slow growth response is cell cycle stage-dependent

Next, the effect of the stage of cell cycle progression on the response to isoleucine withdrawal was investigated.

A culture of highly synchronous young rings was initiated in normal medium. At 3-h intervals, an aliquot of the culture was removed, washed repeatedly to remove any traces of isoleucine, and resuspended in medium lacking isoleucine. After 30 h or 60 h of incubation (for time points > 24 h or < 24 h respectively), the parasitaemia of the cultures was quantified by flow cytometry to determine the relative growth of each sample.

**Fig. 3:**
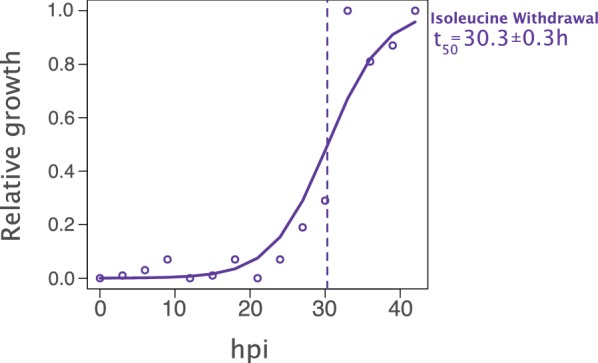
Entry into a growth-retarded state is dependent on the point in the cell cycle at which isoleucine is removed. Synchronous rings were transferred from normal medium to isoleucine-depleted medium every 3 h over a 45-h cycle. The relative growth was quantified by flow cytometry after 30 h (for > 24 hpi) or 60 h (for < 24 hpi) in isoleucine lacking medium. A logistic regression of relative growth vs. hours post invasion (hpi) shows the likelihood of completing the cell cycle increases with hpi (p = 5.85 × 10^−82^, see Additional file 1: Table S1 for regression summary). t50 = the time at which 50% of parasites exhibit growth (vertical broken line)

As expected from earlier experiments, young rings remained at roughly the starting parasitaemia over the course of the incubation. However, as the time of culture increased, a notable increase in the final parasitaemia became evident (Fig. 3). A logistic regression performed on the data confirmed that the likelihood of completing the cell cycle increased as the time of culture in the presence of isoleucine increased (p = 5.85 × 10^−82^, see Additional file 1: Table S1 for regression summary). Based on the logistic model fit to the data, at approximately 30 h post-invasion, 50% of parasites transferred from normal culture medium to isoleucine-lacking medium enter the growth-retarded ‘hibernatory’ state described by Babbitt et al., while the remaining 50% continue the cell cycle at the normal pace and enter the slow-growth state in the subsequent cycle. Beyond 30 h, an increasing proportion of parasites complete the cell cycle uninhibited by the absence of extracellular isoleucine. These data suggest that at around the 30 h into the cell cycle, molecular changes occur to render the parasite refractory to isoleucine withdrawal.

### The isoleucine refractory point coincides with the onset of DNA synthesis

Inferring cellular events in the cell cycle of *P. falciparum* based on time estimates alone can be misleading, as the methods used for synchronization are imperfect, and the in vitro length of the intraerythrocytic cycle has been reported to range anywhere from 38 to 50 h for different parasite isolates and clones [12, 21]. Seeking a better understanding of the basis of refractoriness to isoleucine withdrawal, an attempt was made to link this response to well-established physiological milestones of the cell cycle.

The transition of the parasite from the ring form to the trophozoite form is marked by a dramatic change in the permeability of the host erythrocyte to a range of solutes. This new permeability pathway (NPP) is due to the activation of a parasite-derived or modified host channel in the erythrocyte membrane [22]. Isoleucine is one of the biologically relevant solutes affected by the NPP. While erythrocytes display a basal permeability to isoleucine via the erythrocyte L-system, activation of the NPP increases isoleucine uptake by fivefold [23]. In vitro, NPP activation renders parasites permeable to sorbitol, which leads to rapid lysis of infected erythrocytes with an active NPP (trophozoites and schizonts) but leaves impermeable cells (rings) unharmed. This property provided the basis for a commonly used method for ring-stage synchronization [24]. Given the role of the NPP in isoleucine uptake and the availability of a robust assay of NPP activation, the timing of the isoleucine refractory point relative to NPP activation was determined.

**Fig. 4:**
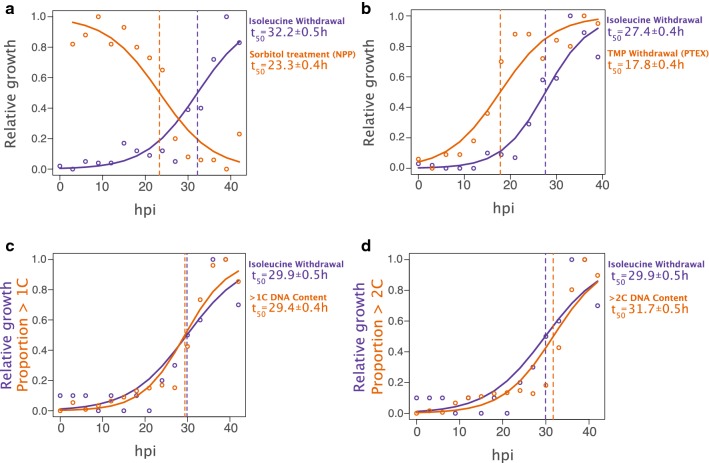
The isoleucine refractory point coincides with the transition into S-phase. **a** The isoleucine refractory point occurs after establishment of the New Permeability Pathway (NPP). The logistic models fit to the data predict the t50 point for sorbitol treatment to be significantly earlier than the isoleucine refractory point (vertical broken lines). **b** The isoleucine refractory point occurs after PTEX-dependent arrest point. Synchronous early rings were incubated in trimethoprim (TMP) to preserve PTEX translocon function and transferred to medium lacking TMP at the indicated times to inhibit PTEX function. Logistic regression models show hpi-dependent increase in relative growth, though the t50 for TMP withdrawal is earlier than the isoleucine refractory point. **c** The isoleucine refractory point overlaps with transition into S-phase. Parasitaemia in isoleucine lacking medium and the proportion of parasites with > 1C DNA content, increase with time. Note that the t_50_ values overlap. When both hours post invasion (hpi) and > 1C DNA content are treated as factors in a model of growth in the absence of extracellular isoleucine, DNA content alone predicts relative growth (p < 5.38 × 10^−12^). **d** The t_50_ point for the transition to > 2C DNA content occurs after the isoleucine refractory point. See Additional file 1: Table S1 for all regression summaries

As before, synchronous parasites in normal medium culture were transferred to medium lacking isoleucine at 3-h intervals across the 45-h life cycle. At each time point, duplicate aliquots of culture were treated with 5% w/v sorbitol and resuspended in normal culture medium for a 72-h recovery period. The relative growth of the two treatments is shown in Fig. 4a. As with isoleucine withdrawal, a logistic regression fit to the sorbitol treated cultures shows a marked time-dependent response. Sorbitol-treated samples display decreased growth with increasing parasite age post-invasion, as would be expected from parasites becoming susceptible to sorbitol lysis as they reach the point of NPP activation (p = 8.73 × 10^−90^). Notably, the logistic model fit to the data maps the 50% relative survival point for sorbitol treatment (23.3 ± 0.4 h) well in advance of the isoleucine refractory point (in this case, 32.2 ± 0.5 h), and finds that sorbitol sensitivity is only a weak predictor of the response to isoleucine removal (p = 0.0136) relative to time of isoleucine removal (p = 1.80 × 10^−28^, Additional file 1: Table S1).

Another molecular milestone in the *P. falciparum* cell cycle is demarcated in late rings by export of certain parasite proteins across the parasitophorous vacuolar membrane into the host erythrocyte by the *Plasmodium* Translocon of Exported Proteins (PTEX) complex. Beck et al. [16], produced a transgenic parasite line in which an essential component of the PTEX complex, Hsp101, was modified such that its activity could be regulated by the chemical ligand trimethoprim (TMP). Removal of TMP from the culture medium leads to inhibition of Hsp101 function, blocking protein export by the PTEX complex. When TMP was removed from the culture at the ring stage, parasites arrested growth as morphological rings, but could resume growth without a loss in viability if the culture was resupplemented with TMP within 48 h. However, if TMP was removed from the culture during the mid-trophozoite phase, the parasites completed the cell cycle unperturbed and reinvaded new cells whereupon they arrested at the late ring stage [16]. This suggests that PTEX-dependent export defines another checkpoint-like event in the cell cycle of *P. falciparum*. This same parasite line was used to determine if the PTEX-dependent checkpoint-like event overlaps with the isoleucine refractory point.

Synchronous young rings were sampled at 3-h intervals across the cell cycle and transferred either to medium lacking isoleucine (but supplemented with TMP), or normal medium lacking TMP. The relative growth of each sample is shown in Fig. 4b. Logistic regression models fit to both treatments show a time-dependent binary response and that the 50% growth point for PTEX-dependent growth-arrest (17.8 ± 0.4 h) is well ahead of the isoleucine-dependent growth-retardation point (here 27.4 ± 0.4 h). Like sorbitol sensitivity, PTEX-dependent growth arrest is a weak predictor of isoleucine refractoriness (p = 0.00338) relative the point in the cell cycle at which extracellular isoleucine is removed (p = 5.59 × 10^−44^, Additional file 1: Table S1). The 3D7 Hsp101-DD clone used for this assay displays a shorter cell cycle time than the NF54 clone, which likely contributes to the somewhat earlier timing the isoleucine response point. While it is difficult to project the PTEX-arrest point onto the sorbitol-NPP point calculated above with the NF54 clone, Beck et al. [16] demonstrated that PTEX-arrested parasites were resistant to sorbitol treatment, placing activation of the NPP after the PTEX-dependent arrest point.

Next, the mapping of the isoleucine refractoriness point relative to the transition into S-phase and schizogony was tested. Synchronous young rings were transferred from normal medium into isoleucine-depleted medium at 3-h intervals. At each time point, aliquots were fixed, permeabilized, and stained with SYBR GREEN I for DNA content analysis by flow cytometry (Fig. 4c). A logistic regression model fit to the proportion of parasites with > 1C DNA content overlaps with the proportion of parasites continuing normal growth, with 50% of the parasites transitioning from 1C DNA content to > 1C content (29.9 ± 0.5 h) at a point indistinguishable from the point at which 50% of the parasites become refractory to isoleucine withdrawal (29.4 ± 0.4 h). In fact, when a regression model was fit with both age post-invasion and proportion of > 1C parasites as factors, the DNA content alone predicts the response of the parasites to isoleucine (p = 5.38 × 10^−12^ as compared to p = 0.577 for hour of Ile removal alone, Additional file 1: Table S1). From the same data, the timing of the transition from < 2C to > 2C DNA content relative to the isoleucine-dependent point was also tested. While temporally close, the transition from < 2C to > 2C (31.7 ± 0.5 h) occurs after cells have become refractory to the removal of isoleucine (Fig. 4d). The transition from below 2C to greater than 2C DNA content does not predict the response to isoleucine removal (p = 0.341, Additional file 1: Table S1).

## Discussion

**Fig. 5:**
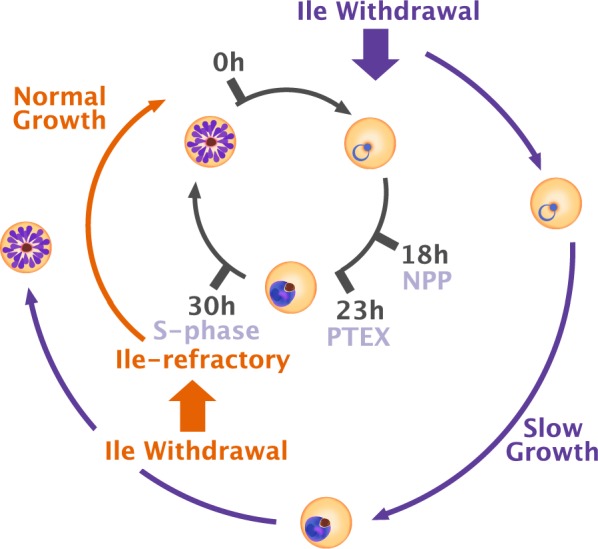
*Plasmodium falciparum* enters a cell cycle stage-dependent slow growth program upon extracellular isoleucine removal. If isoleucine is removed in the early stages of the cell cycle, *P. falciparum* enters a state of slow growth in which it can complete schizogony and reinvade new erythrocytes. If isoleucine is removed after approximately 30 h post-invasion, the parasite continues schizogony at the normal rate of growth. This refractory point coincides with the onset of S-phase and occurs well after establishment of the New Permeability Pathway (NPP) and the PTEX-dependent growth arrest point

This work showed that when deprived of extracellular isoleucine, *P. falciparum* enters a reversible state of slow growth in which it progresses through the cell cycle at approximately half its normal pace (Fig. 5). The parasite can complete the cell cycle and invade erythrocytes anew in the complete absence of isoleucine in the medium. However, if isoleucine is removed after DNA replication has commenced, the parasite does not enter the slow program and instead continues its mitotic cycle at the normal rate.

Similar phenomena have been described in other well-studied eukaryote systems. In the yeast *Saccharomyces cerevisiae*, cells deprived of nitrogen or glucose in early G1 will grow slowly and enter a quiescent state. However, if the nutrient is removed after a specific point late in G1, cells proceed with S-phase and mitosis, then enter the quiescent state in the subsequent generation [25]. A similar process occurs in yeast for differentiation into budding cells when stimulated with mating factor [26], and an analogous phenomenon exists in mammalian cells starved of growth factors or amino acids [10, 27]. In these organisms, “the point of no return” at which cells commit to either growth arrest or mitosis is referred to as a G1 checkpoint.

In yeast and mammalian cells, the G1 checkpoint is determined at the molecular level through the interaction of cyclins and cyclin-dependent kinases (CDKs), which activate a positive feedback loop of S-phase transcription factors, irreversibly driving the cell cycle forward. The observed *P. falciparum* isoleucine refractory point may be determined by a similar mechanism. The *P. falciparum* genome encodes three or four putative cyclins and five kinases of the CDK family, all of which display periodic expression profiles across the intra-erythrocytic cycle. Two CDKs (PF3D7_1014400, PF3D7_1014400), and a single cyclin (PF3D7_1463700), display changes from high expression in G1 to low expression in schizogony, with an inflection point at roughly 30 h post invasion [28], making them potential candidate regulators of the G1/S transition. It will be interesting to determine whether manipulation of expression or stability of these candidate proteins affects the timing of the isoleucine refractory point and entrance into S-phase.

It remains unknown how *P. falciparum* senses and initiates the slow growth response to isoleucine withdrawal. Babbitt et al. reported that the *Pf*eIK1, the orthologue of the eukaryote GCN2 uncharged tRNA-sensing kinase, rapidly phosphorylates eIF2α in response to isoleucine removal but has no effect on observed growth response or survival [12]. As previously reported, *PfMaf1,* an orthologue of a highly conserved eukaryote gene associated with starvation responses in other organisms, is required for recovery from isoleucine withdrawal, but has no effect on initiation of slow growth [29]. A recent study demonstrated that the murine model parasite *Plasmodium berghei* decreases the number of merozoites per schizont in mice fed a calorie-restricted diet due to the activity of *PbKIN*, a kinase conserved in the *Plasmodium* genus showing structural similarities to the eukaryote AMPK family of genes involved in the sensing of cellular ATP levels [30]. It is not clear whether caloric restriction slows the *P. berghei* cell cycle progression in a manner analogous to what has been observed here with *P. falciparum* in vitro. Mouse haemoglobin contains isoleucine, precluding isoleucine withdrawal experiments in that model system.

While initial studies of artemisinin-resistant parasites in the field suggested a mechanism of programmed dormancy, subsequent follow-up experiments in vitro have led to more nuanced models. Artemisinin appears to induce a transient slowing of the parasite cell cycle [31], like amino acid withdrawal, which is likely the source of the persistent ring forms observed in clinical studies. Parasites harbouring mutations associated with artemisinin resistance display higher levels of survival to artemisinin insult and more robust recovery from the drug-induced growth-retardation [32]. Intriguingly, in vitro data suggests that artemisinin insult, like isoleucine withdrawal, triggers eIF2α phosphorylation [33]. Unlike isoleucine deprivation, however, eIF2α phosphorylation appears to be necessary for recovery [33]. The extreme increase in artemisinin potency upon the onset of haemoglobin digestion [2] precludes the study of cell cycle-stage dependent effects of the drug on growth retardation in the manner performed here. In the future, when the genetic basis for the resistance phenotype is better understood, it will be interesting to learn whether parasites also become refractory to drug-induced growth retardation once the G1 to S-phase threshold is crossed.

## Conclusions

When deprived of isoleucine in vitro, pre-S-phase *P. falciparum* parasites dramatically slow progression through the cell cycle. However, if isoleucine is withdrawn after the onset of S-phase, the parasites continue progression at a normal pace. This behaviour demarcates a previously undescribed nutrient-sensitive point in *P. falciparum* cell cycle that has important implications for how the parasite is able to adapt its growth rate in response to environmental changes.

## Supplementary information

**Additional file 1: Table S1.** Regression summaries.

## Data Availability

Data sharing is not applicable to this article as no datasets were generated or analysed during the current study.

## Abbreviations

CDK: Cyclin dependent kinase
hpi: Hours post invasion
Ile: Isoleucine
NPP: New permeability pathway
PBS: Phosphate buffered saline
PTEX: *Plasmodium* translocon of exported proteins
RPMI: Roswell Park Memorial Institute 1640
TMP: Trimethoprim
