# Supplementary material for: The response of Plasmodium falciparum to isoleucine withdrawal is dependent on the stage of progression through the intraerythrocytic cell cycle
Source: Malar J. 2020 Apr 8;19:147. doi: 10.1186/s12936-020-03220-w (PMC7140564; doi:10.1186/s12936-020-03220-w)
Supplement: Supplementary file 1 — Additional file 1: Table S1. Regression summaries. [file 12936_2020_3220_MOESM1_ESM.docx]

Table S1: **Regression Summaries**

| **Figure reference** | **Generalized Linear Model** |  | **Coefficient** | **Estimate** | **Std Error** | **p-value** |
| --- | --- | --- | --- | --- | --- | --- |
|  |  |  |  |  |  |  |
| Figure 3 | regrowth ~ hour of ile removal | β0 | intercept | -4.456744 | 0.22586 | 1.14E-86 |
|  |  | β1 | ile removal (h) | 0.149284 | 0.007785 | 5.85E-82 |
|  |  |  |  |  |  |  |
|  |  |  |  |  |  |  |
|  |  |  |  |  |  |  |
| Figure 4A | regrowth ~ sorbitol treatment | β0 | intercept | 3.7560399 | 0.198859292 | 1.43E-79 |
|  |  | β1 | sorbitol treatment (h) | -0.1609643 | 0.008011506 | 8.73E-90 |
|  |  |  |  |  |  |  |
| Figure 4A | regrowth ~ hour of ile removal + sorbitol treatment | β0 | intercept | -6.79896682 | 0.669022708 | 2.91E-24 |
|  |  | β1 | ile removal (h) | 0.20374746 | 0.018409162 | 1.80E-28 |
|  |  | β2 | sorbitol treatment (h) | 0.01170564 | 0.004743388 | 0.0136 |
|  |  |  |  |  |  |  |
|  |  |  |  |  |  |  |
| Figure 4B | regrowth ~ TMP removal | β0 | intercept | -3.1579101 | 0.175719768 | 3.27E-72 |
|  |  | β1 | TMP removal (h) | 0.1777227 | 0.008793601 | 7.92E-91 |
|  |  |  |  |  |  |  |
| Figure 4B | regrowth ~ hour of Ile removal + TMP removal | β0 | intercept | -5.51460144 | 0.30462267 | 3.01E-73 |
|  |  | β1 | ile removal (h) | 0.25204654 | 0.01812125 | 5.59E-44 |
|  |  | β2 | TMP removal (h) | -0.01816137 | 0.00619636 | 0.003379019 |
|  |  |  |  |  |  |  |
|  |  |  |  |  |  |  |
| Figure 4C | regrowth ~ DNA content >1C | β0 | intercept | -2.654435 | 0.1163768 | 3.74E-115 |
|  |  | β1 | >1C DNA | 5.080719 | 0.2317925 | 1.70E-106 |
|  |  |  |  |  |  |  |
| Figure 4D | regrowth ~ DNA content > 2C | β0 | intercept | -2.351025 | 0.1021103 | 2.66E-117 |
|  |  | β1 | >2C DNA | 5.429859 | 0.2845587 | 3.59E-81 |
|  |  |  |  |  |  |  |
| Figure 4C & 4D | regrowth ~ hour of ile removal + >1C + > 2C | β0 | intercept | -2.767775 | 0.214704 | 5.05E-38 |
|  |  | β1 | ile removal (h) | 0.007705 | 0.013832 | 0.577 |
|  |  | β2 | >1C DNA | 5.412561 | 0.784967 | 5.38E-12 |
|  |  | β3 | >2C DNA | -0.66502 | 0.698742 | 0.341 |
